# Procedure efficiency and diagnostic outcomes during the learning curve of transperineal MRI‐guided prostate biopsy

**DOI:** 10.1002/bco2.70237

**Published:** 2026-07-08

**Authors:** Mario Hallaschek, Jeremy Kwe, Friedrich Helke, Ivan Platzek, Katharina Böhm, Martin Baunacke, Angelika Borkowetz, Christian Thomas, Roman Herout, Benedikt Hoeh

**Affiliations:** ^1^ Department of Urology University Hospital Carl Gustav Carus, TUD Dresden University of Technology Dresden Germany; ^2^ Department of Radiology University Hospital Carl Gustav Carus, TUD Dresden University of Technology Dresden Germany; ^3^ Department of Urology University Medical Centre Rostock, University Medicine Rostock Rostock Germany

**Keywords:** clinically significant prostate cancer, learning curve, MRI‐guided biopsy, transperineal prostate biopsy

## Abstract

**Objectives:**

This work aimed to investigate the role of surgeon experience on operating time and detection rate of clinically significant prostate cancer (csPCa) in a large contemporary cohort undergoing transperineal (TP) MRI‐targeted prostate biopsy.

**Materials and Methods:**

We retrospectively reviewed patients who underwent TP MRI‐guided prostate biopsies between 10/2018 and 04/2024. Patients with suspicious MRI lesions (PI‐RADS 3, 4 and 5) formed the study cohort. All patients underwent MRI‐targeted biopsies of suspicious lesions combined with a standard 12‐core systematic biopsy. Caseloads—both total and in increments of 50 cases—were calculated for each surgeon. We employed univariable and multivariable Poisson regression models to analyse biopsy duration, and logistic regression models to assess csPCa detection.

**Results:**

Out of 3336 MRI‐guided TP biopsies, 1148 (34%) involved PI‐RADS 3, 1398 (42%) PI‐RADS4 and 790 (24%) PI‐RADS 5 as index lesions. Most patients (55%) had one suspicious lesion. The median caseload per surgeon was 63 (IQR [interquartile range]: 30–110). Biopsy duration significantly decreased after the first 50 procedures (13 min [95% CI: 10–18] to 10 min [95% CI: 8–15]) and plateaued thereafter. Multivariable Poisson regression demonstrated increased caseload independently associated with shorter biopsy duration (IRR 0.891 [95% CI: 0.833–0.899], *p* < 0.001). The overall csPCa detection rate was 43%, with no significant correlation to caseload. These findings were consistent across subgroup analyses based on PI‐RADS score.

**Conclusions:**

This large, contemporary study demonstrated that biopsy duration decreased significantly after approximately 50 cases, indicating a learning curve without compromising the detection rate of csPCa.

## 1 INTRODUCTION

Prostate cancer is the second most common cancer in men worldwide. In 2022, 74 895 new cases were recorded in Germany, corresponding to an age‐standardized incidence rate of 108.5 per 100 000 men.^1^ Since multiparametric magnetic resonance imaging (mpMRI) was integrated into the diagnostic algorithm for prostate cancer, it has changed fundamentally. Until recently, transrectal (TR) MRI‐guided prostate biopsy combining targeted and systematic biopsies was considered the gold standard for histological confirmation in patients with elevated prostate‐specific antigen (PSA) levels. However, the transperineal (TP) approach has gained importance. Several studies have demonstrated lower infection rates and comparable detection rates (DRs) of clinically significant prostate cancer (csPCa) using the TP technique.^2^, ^3^, ^4^, ^5^ Evidence on the TR approach has shown a learning curve (LC), with both efficiency and quality improving as surgeons' experience and case volume increase.^2^, ^6^, ^7^ In contrast, current data on the LC for MRI‐guided TP prostate biopsy are limited and mostly influenced by small case series or variations in case mix.^8^, ^9^, ^10^, ^11^ It is important to identify a LC to ensure high quality standards on patient safety and efficiency.^12^ This study therefore aimed to evaluate the LC for MRI‐guided TP prostate biopsy, focusing on procedural efficiency and diagnostic outcomes over caseload among surgeons.

## 2 MATERIAL AND METHODS

### 2.1 Study population

Following approval of the institutional review boards of the Ethic committee of the Technische Universität Dresden, patients who obtained a TP MRI‐guided prostate biopsy between 10/2018 and 04/2024 were retrospectively identified within a prospectively maintained database. Subsequently, patients undergoing solely systematic prostate biopsy as well as patients with unknown PI‐RADS information were excluded. Solely patients with at least one suspicious lesion, defined as PI‐RADS 3, PI‐RADS 4 or PI‐RADS 5, according to the Prostate Imaging Reporting & Data System (version 2.0 or more recent) were included within the final study cohort.^13^ All patients underwent MRI‐targeted biopsies of suspicious lesions combined with a standard 12‐core systematic biopsy, in accordance with institutional protocols. The index lesion was defined as the target lesion with the highest PI‐RADS score. In case of two or more target lesions with the same PI‐RADS score, the index lesion was defined based on the maximal diameter. T2‐weighted imaging was used for contouring the prostate and target lesions, and then a 3‐D model of the prostate and target lesions was built using the bkFusion (BK Medical, Herlev, Denmark) system. Due to differences in MRI protocols and quality between internal (Institute for Diagnostic and Interventional Radiology at the Technische Universität Dresden) and external imaging facilities, MR‐Imaging was reviewed by dedicated genitourinary radiologists and lesions were marked accordingly prior to biopsy. In case of multiple lesions, MRI‐guided biopsies covered all suspicious lesions separately. A minimum of at least two cores was taken per lesion based on size. MRI‐guided biopsies were performed in a dorsal lithotomy position under local anaesthesia or anaesthesia. Following institutional protocol, an antibiotic prophylaxis, if not otherwise indicated, was administered perioperatively. The operating time (OT) began when local anaesthesia was administered to both plexuses, with the same reference point applied to patients under general anaesthesia.

CsPCa was defined as Gleason Score ≥3 + 4 or ISUP grade group ≥2 in final biopsy pathology report.^14^ Caseload calculations were based solely on MRI‐guided TP biopsy experience, irrespective of previous TR or systematic‐only TP biopsy experiences.

### 2.2 Statistical analysis

The statistical analyses consisted of the following steps: First, patient and tumour characteristics of the overall study population, irrespective of PI‐RADS index lesion or surgeon's caseload, were tabulated. Descriptive statistics included frequencies and proportions for categorical variables. Medians and interquartile ranges (IQR) were reported as continuously coded variables. The chi‐square test examined the statistical significance of the differences in proportions while the Kruskal–Wallis test was used to examine differences in medians.

Second, a scatter plot with a LOESS (Locally Estimated Scatterplot Smoothing) smoothed trend line was utilized to illustrate the relationship between caseload and biopsy duration. Subsequently, two separate univariable and multivariable generalized linear models (GLMs) utilizing a Poisson family with a log link function were fitted to investigate the predictor status of caseload on biopsy duration (log (caseload + 1) versus categorically coded [0–50 vs. 51–100 vs. 101–150 vs. ≥151]). Adjustment variables consisted of: prostate volume (continuously coded), age at biopsy (continuously coded), number of PI‐RADS lesions (continuously coded), history of TR prostate biopsy experience (no vs. yes), and initial biopsy (no vs. yes).

Third, DRs of csPCa were calculated within the overall cohort and subsequently according to surgeons' caseload (0–50 vs. 51–100 vs. 101–150 vs. ≥151).

Hereafter, to test for a relation between csPCa prevalence and caseload (categorically coded [0–50 vs. 51–100 vs. 101–150 vs. ≥151]), univariable and multivariable logistic regression models were fitted relying on the following variables: prostate volume (continuously coded), prostate‐specific antigen (PSA, ng/mL continuously coded), digital rectal examination (normal vs. suspicious vs. unknown), age at biopsy (continuously coded), number of PI‐RADS lesions (continuously coded), index PI‐RADS lesion (3 vs. 4 vs. 5), history of TR prostate biopsy experience (no vs. yes) and history of prostate cancer (no vs. yes).

Fourth and finally, subgroup analyses were performed for patients with index lesion of PI‐RADS 3, PI‐RADS 4 or PI‐RADS 5, respectively. Here, univariable and multivariable logistic regression models were refitted relying on the aforementioned variables. All tests were two‐sided with a level of significance set at *p* < 0.05, and R‐software environment for statistical computing and graphics (version 3.4.3) was used for all analyses.^15^

## 3 RESULTS

This retrospective monocentric study examined patients at the Department of Urology at Carl Gustav Carus University Hospital Dresden who underwent an MRI‐guided prostate biopsy between October 2018 and April 2024. During this period, 3336 patients fulfilled the aforementioned inclusion criteria and thus were included in the study.

As seen in Table 1, the median age was 68 years (IQR 62–73) and the median PSA was 7.2 ng/mL (IQR 5.2–10.7). Prostate cancer had previously been detected in 16% of patients (*n* = 533) and suspicious findings were observed during digital rectal examination in 15% of patients (*n* = 510). The median prostate volume was 50 mL (IQR 37–74), resulting in a median PSA density of 0.14 ng/mL/cm^3^ (IQR 0.09–0.22).

**TABLE 1:** Patient and tumour characteristics of MRI‐guided transperineal prostate biopsy between 10/2018 and 04/2024. All values are median (interquartile range) or frequencies (%).

	Overall, *N* = 3336	Caseload: 1–50, *N* = 1367 (41%)	Caseload: 51–100, *N* = 1005 (30%)	Caseload: 101–150, *N* = 540 (16%)	Caseload: ≥150, *N* = 424 (13%)	*p* value
Age at biopsy [years]	68 (62, 73)	68 (62, 73)	67 (62, 73)	67 (61, 72)	68 (63, 73)	0.3
Prostate‐specific antigen [ng/mL]	7.2 (5.2, 10.7)	7.3 (5.3, 10.7)	7.1 (5.2, 10.7)	7.4 (5.4, 10.4)	7.0 (4.9, 10.7)	0.4
Prostate volume [cc]	50 (37, 74)	50 (36, 72)	52 (38, 75)	51 (40, 74)	50 (37, 71)	0.3
PSA density [(ng/mL)/cc]	0.14 (0.09, 0.22)	0.14 (0.09, 0.23)	0.13 (0.09, 0.22)	0.13 (0.09, 0.21)	0.14 (0.09, 0.21)	0.5
Caseload per surgeon	64 (30, 110)	24 (12, 37)	73 (61, 86)	122 (110, 135)	185 (167, 209)	<0.001
History of prostate cancer						0.7
No	2802 (84%)	1146 (84%)	842 (84%)	462 (86%)	352 (83%)	
Yes	533 (16%)	221 (16%)	162 (16%)	78 (14%)	72 (17%)	
Naive prostate biopsy						0.004
No	1803 (54%)	775 (57%)	550 (55%)	277 (51%)	201 (47%)	
Yes	1533 (46%)	592 (43%)	455 (45%)	263 (49%)	223 (53%)	
Digital rectal examination						0.019
Normal	2550 (77%)	1055 (77%)	746 (74%)	428 (79%)	321 (76%)	
Suspicious	510 (15%)	216 (16%)	149 (15%)	73 (14%)	72 (17%)	
Unknown	273 (8.2%)	96 (7.0%)	108 (11%)	39 (7.2%)	30 (7.1%)	
No. of MRI‐based lesions						
1	1827 (55%)	726 (53%)	541 (54%)	314 (58%)	246 (58%)	
2	1124 (34%)	471 (34%)	344 (34%)	171 (32%)	138 (33%)	
3	335 (10%)	145 (11%)	102 (10%)	50 (9.3%)	38 (9.0%)	
≥4	49 (1.5%)	24 (1.8%)	18 (1.8%)	5 (0.9%)	2 (0.5%)	
Index PI‐RADS lesion						0.3
3	1148 (34%)	447 (33%)	363 (36%)	193 (36%)	145 (34%)	
4	1398 (42%)	573 (42%)	411 (41%)	223 (41%)	191 (45%)	
5	790 (24%)	347 (25%)	231 (23%)	124 (23%)	88 (21%)	
Transrectal prostate biopsy experience						<0.001
No	1564 (47%)	555 (41%)	415 (41%)	252 (47%)	342 (81%)	
Yes	1772 (53%)	812 (59%)	590 (59%)	288 (53%)	82 (19%)	

Abbreviations: No., number; MRI, magnetic resonance imaging; PI‐RADS, Prostate Imaging Reporting & Data System; PSA, prostate‐specific antigen.

In 55% of cases (*n* = 1827), mpMRI showed one suspicious lesion; in 34% (*n* = 1124) two suspicious lesions; in 10% (*n* = 335) three suspicious lesions and in 1.5% (*n* = 49) more than three suspicious lesions. The distribution of index lesions according to PI‐RADS was as follows: 34% (*n* = 1148) were PI‐RADS 3, 42% (*n* = 1398) were PI‐RADS 4, and 24% (*n* = 790) were PI‐RADS 5.

A total of 31 surgeons performed prostate biopsies. To analyse the biopsy results further in relation to the experience of the surgeon, we defined four subgroups: 1–50 biopsies, 51–100 biopsies, 101–150 biopsies and more than 150 biopsies. The median caseload per surgeon was 64 biopsies (IQR 30–110).

**FIGURE 1:**
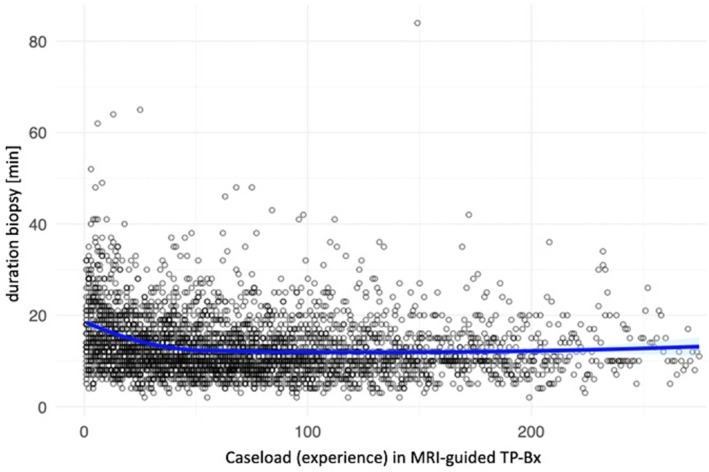
Scatter plot with a LOESS (locally estimated scatterplot smoothing) smoothed trend line depicting the relationship between caseload and operating time (biopsy duration) among 3336 cases undergoing MRI‐guided transperineal prostate biopsy between 10/2018 and 04/2024.

As seen in Table S1, no significant differences were found between the respective case number groups when the basic data was examined. Considering all case numbers, the median OT was 11 min (IQR 9–16). As shown in Figure 1, OT decreased significantly with increasing caseload, from a median of 13 min (IQR 10–18) initially to 10 min (IQR 9–15) for caseloads exceeding 50 biopsies per surgeon, with no further reduction thereafter (*p* < 0.001).

Using generalized linear models, we examined the effect of surgeon caseload on biopsy duration. As presented in Table 2, biopsy time significantly decreased with increasing caseload in both the continuous, log‐transformed analysis and categorical analysis by case number group. In both univariable and multivariable models, a significant reduction in the incidence rate ratio (IRR) was observed. The logistic regression yielded an IRR of 0.890 (95% CI 0.882–0.898), while the multivariable analysis using surgeons with ≤50 cases as the reference showed IRRs of 0.831 (95% CI 0.813–0.850), 0.834 (95% CI 0.810–0.857) and 0.830 (95% CI 0.804–0.857) for caseload groups of 51–100, 101–150 and ≥151, respectively (*p* < 0.001). A higher prostate volume, the presence of multiple lesions on MRI, and the absence of prior biopsy resulted in an IRR > 1, indicating a significant increase in biopsy time in both univariable and multivariable analysis.

**TABLE 2:** Two separate generalized linear models (GLMs) investigating the predictor status of caseload (log (caseload + 1) vs. categorically [0–50 vs. 51–100 vs. 101–150 vs. ≥151]), patient and biopsy characteristics on operating time among 3336 cases treated with MRI‐guided transperineal prostate biopsy.

	Univariable GLM I.			Multivariable GLM I.		
	IRR	95% CI	*p* value	IRR	95% CI	*p* value
Log (caseload + 1)	0.889	0.882, 0.897	<0.001	0.890	0.882, 0.898	<0.001
Age	1.001	1.000, 1.002	0.064	1.001	0.999, 1.002	0.400
Prostate volume	1.001	1.000, 1.001	<0.001	1.001	1.001, 1.001	<0.001
Number MRI lesions						
1	Ref.			Ref		
2	1.074	1.052, 1.096	<0.001	1.062	1.041, 1.084	<0.001
3	1.147	1.112, 1.183	<0.001	1.124	1.089, 1.159	<0.001
≥4	1.210	1.124, 1.300	<0.001	1.173	1.089, 1.260	<0.001
Naive biopsy (Ref. no)	1.021	1.001, 1.040	0.035	1.045	1.025, 1.065	<0.001
Experience in transrectal biopsy (ref. no)	1.018	0.999, 1.038	0.061	0.984	0.965, 1.003	0.083

	Univariable GLM II.			Multivariable GLM II.		
	IRR	95% CI	*p* value	IRR	95% CI	*p* value
Caseload 1–50	Ref.			Ref.		
51–100	0.832	0.813, 0.851	<0.001	0.831	0.813, 0.850	<0.001
101–150	0.827	0.804, 0.850	<0.001	0.834	0.810, 0.857	<0.001
≥151	0.827	0.802, 0.853	<0.001	0.830	0.804, 0.857	<0.001
Age	1.001	1.000, 1.002	0.064	1.001	1.048, 1.092	<0.001
Prostate volume	1.001	1.000, 1.001	<0.001	1.001	1.000, 1.001	<0.001
Number MRI lesions						
1	Ref.			Ref.		
2	1.074	1.052, 1.096	<0.001	1.070	1.048, 1.092	<0.001
3	1.147	1.112, 1.183	<0.001	1.133	1.098, 1.169	<0.001
≥4	1.210	1.124, 1.300	<0.001	1.185	1.101, 1.274	<0.001
Naive biopsy (ref. no)	1.021	1.001, 1.040	0.035	1.041	1.021, 1.061	<0.001
Experience in transrectal biopsy (ref. no)	1.018	0.999, 1.038	0.061	1.002	0.983, 1.022	0.836

Abbreviations: 95% CI, 95% Confidence Interval; GLM, general linear model; IRR, incidence rate ratio.

The MRI‐guided prostate biopsy confirmed the presence of csPCa in 43% of patients (*n* = 1413). The DR did not vary significantly depending on the number of biopsies performed (*p* = 0.8). The DR was 43% (*n* = 585) for 0–50 biopsies, 41% (*n* = 412) for 51–100 biopsies, and 43% for 101–150 biopsies (*n* = 233) and more than 150 biopsies (*n* = 180).

Univariable and multivariable logistic regression analyses revealed that the DR did not vary significantly depending on the number of biopsies performed when the first 50 biopsies were used as a reference point. The odds ratios for the multivariable analysis were 1.057 (95% CI 0.867–1.288, *p* = 0.583), 1.142 (95% CI 0.897–1.454, *p* = 0.281) and 0.958 (95% CI 0.729–1.257, *p* = 0.757) for caseloads of 51–100, 101–150 and ≥151, respectively (Table 3).

**TABLE 3:** Univariable and multivariable logistic regression models investigating the predictor status for detection of clinically significant prostate cancer according to caseload among 3336 cases treated with MRI‐guided transperineal prostate biopsy.

	Univariable				Multivariable^a^			
	Odds ratio	2.5%	97.5%	*p* value	Odds ratio	2.5%	97.5%	*p* value
Caseload 1–50	Ref.				Ref.			
51–100	0.9312	0.7881	1.0999	0.4019	1.0569	0.8674	1.2877	0.5831
101–150	0.9993	0.8152	1.2241	0.9948	1.1422	0.8970	1.4540	0.2806
≥151	0.9764	0.7816	1.2182	0.8329	0.9579	0.7289	1.2573	0.7569

^a^ Adjusted for: age, digital rectal examination, number of PI‐RADS lesions, index PI‐RADS lesion, PSA at biopsy, prostate volume, history of prostate cancer, experience in transrectal prostate biopsy.

A subgroup analysis considering the radiological classification revealed csPCa in 20% of PI‐RADS 3 lesions (*n* = 219), 46% of PI‐RADS 4 lesions (*n* = 628) and 71% of PI‐RADS 5 lesions (*n* = 549). There was no significant difference in DR according to the surgeon's experience (*p* = 0.8–0.9). Again, multivariable logistic regression analysis revealed that the DR did not vary significantly depending on the number of biopsies performed when the first 50 biopsies were used as a reference point (Table S2).

## 4 DISCUSSION

In recent years, the TP approach has become the preferred method for MRI‐guided prostate biopsy.^2^, ^3^, ^11^, ^14^ Previous studies examined the LC of either a single surgeon or a small group of surgeons with mostly a case mix of TP and TR prostate biopsies. Consequently, their findings can only be applied to a limited extent when defining the LC within the TP approach.^12^ The current study aimed to investigate the LC of TP MRI‐guided prostate biopsies within a large, contemporary cohort of patients.

First, the present study demonstrated a significant reduction in OT with increasing biopsy experience. After 50 biopsies, median OT plateaued at 10 min (IQR 9–15), likely due to an optimized procedure driven by greater procedural familiarity. When comparing data, it should be noted that OT is influenced by different biopsy systems and protocols, as well as the number of punctured lesions. Falkenbach et al. observed a comparable steady state in OT after 50 biopsies, but they reported a longer OT of 27 min (IQR 22–32). This difference may be due to protocol variations that incorporated MRI segmentation and lesion targeting into OT.^11^ Halstuch et al. reported a decrease in OT from 55 to 18 min, reaching a plateau after 124 biopsies. In their study, a single urologist performed both approaches, initially TR and subsequently TP prostate biopsies, suggesting a potential influence between the two techniques.^9^ Meanwhile, Ramacciotti et al. reported a reduction in OT from 46 to 19 min for freehand TP MRI‐guided prostate biopsy, with a plateau after 156 procedures. These findings came from a single urologist with extensive prior experience in the TR approach (>1500 TR MRI‐guided biopsies).^16^

Second, the present study demonstrated that the DR for csPCa did not differ significantly with the surgeon's caseload. The overall DR for csPCa was 43% (*n* = 1410). Multivariable regression analysis revealed no association between the surgeon's caseload and the DR of csPCa. To identify potential differences in the DR across individual index lesions, subgroup analyses were performed after stratification according to index lesion. Here, the DR for PI‐RADS 3, 4 and 5 index lesions were 20%, 46% and 71%, respectively. In line with findings based on the overall cohort, a different set of multivariable regression analyses were conducted and demonstrated that the surgeon's caseload was not statistically associated with the detection of csPCa. Xu et al. used cumulative sum analysis (CUSUM) to identify a turning point in the LC after 52 biopsies. Opposed to our observations, the DR for PI‐RADS 4 lesions increased considerably from 26.9% to 61.3% with increasing caseload per surgeon whereas the DR in the current study was already substantially higher from the very beginning.^10^ Halstuch et al. also reported a significant increase in the DR of csPCa with increasing biopsy experience. They observed an increase in the DR in PI‐RADS 3 lesions from 30% to 55%. A stable DR was only achieved after 130 biopsies. However, these findings may have been influenced by the study design as well as differences in patient and tumour characteristics.^9^ Finally, Falkenbach et al. examined the LC after switching from TR to TP MRI‐guided biopsy in a small patient cohort. A categorical comparison revealed a modest difference in the DR between the learning phase (DR 56.3% for ≤100 prostate biopsies) and subsequent biopsies (DR 64.4%).^11^ However, the study by Falkenbach et al. relied on a small cohort of only 200 TP biopsies in comparison to 3336 TP biopsies included within the current study and thus, comparison is limited.

An effective and standardized approach at our institution could be discussed as the reason for the high‐quality results throughout the entire learning phase. All new surgeons were accompanied by an experienced mentor for their first biopsies, and all MRIs, irrespectively of whether performed internally or externally, were reviewed and lesions marked by a board‐certified radiologist with extensive experience in prostate MRI interpretation. Furthermore, the current study was performed at a single centre using the same biopsy system. Taking together, the current results indicate that using a standardized institutional protocol as well as a mentoring program can help maintain the efficiency and quality of TP MRI‐guided prostate biopsies during the critical learning phase. We observed a significant decline in OT within the first 50 biopsies while demonstrating stable DR of csPCa, regardless of the surgeon's experience.

Despite the noteworthy findings of the current study, several limitations must be acknowledged. The retrospective design might include potential selection bias and imprecise procedure time measurements. Additionally, the results may have been partially influenced by the initial group of surgeons, who had prior experience with TR biopsies. This may have influenced the results towards shorter biopsy time and a higher DR from the beginning. However, after multivariable adjustment for previous TR biopsy experience, the results remained unchanged. Another limitation is the absence of data collection regarding postoperative pain and postoperative complications. Furthermore, variability in the quality of MRIs obtained from external institutions could not be fully controlled, although each scan was reviewed and PI‐RADS lesions were marked by an experienced in‐house radiologist. Within the study cohort, the vast majority of patients were treated under local anaesthesia; however, some patients received additional sedation for the MRI‐guided biopsy. Even though the vast majority of patients were treated with local anaesthesia, the potential effect of sedation cannot be excluded and the results should be interpreted accordingly. The single‐centre design limits the study's generalizability to other institutions with different equipment or protocols. Nevertheless, due to the large, contemporary cohort and inclusion of data from multiple surgeons, the findings are likely more generalizable than those of previously published studies.

## 5 CONCLUSIONS

This large, contemporary study demonstrated that the duration of TP MRI‐guided prostate biopsies decreased significantly after approximately 50 cases, reaching a plateau thereafter. This indicates a LC. Regarding the quality of the procedure, there was no difference in DR of csPCa with increasing surgeon experience. In summary, a standardized institutional protocol consisting of professional interdisciplinary collaboration and dedicated onboarding training is key to achieving effectiveness and high quality during the learning phase of a surgical procedure.

## AUTHOR CONTRIBUTIONS

All authors contributed substantially to the study conception and design. **Mario Hallaschek**: Formal analysis; original draft preparation. **Jeremy Kwe**: Data acquisition. **Friedrich Helke**: Data acquisition. **Ivan Platzek**: Data acquisition; writing—reviewing and editing. **Katharina Boehm**: Writing—reviewing and editing. **Martin Baunacke**: Writing—reviewing and editing. **Angelika Borkowetz**: Writing—reviewing and editing. **Christian Thomas**: Conceptualization; writing—reviewing and editing. **Roman Herout**: Conceptualization; writing—reviewing and editing; supervision. **Benedikt Hoeh**: Conceptualization; writing—reviewing and editing; supervision.

## CONFLICT OF INTEREST STATEMENT

The authors declare that there is no conflict of interest.

## Supporting information


**Table S1.** Perioperative and oncological outcomes of MRI‐guided transperineal prostate biopsy between 10/2018 and 04/2024. All values are median (Interquartile range) or frequencies (%).

**Table S2.** Separate univariable and multivariable logistic regression models investigating the predictor status for detection of clinically significant prostate cancer according to caseload among 3336 cases treated with MRI‐guided transperineal prostate biopsy stratified according to index PI‐RADS lesion (PI‐RADS 3, PI‐RADS 4, PI‐RADS 5).

## Data Availability

All datasets generated for this study are included in the manuscript.
