# Supplementary material for: Procedure efficiency and diagnostic outcomes during the learning curve of transperineal MRI‐guided prostate biopsy
Source: BJUI Compass. 2026 Jul 8;7(7):e70237. doi: 10.1002/bco2.70237 (PMC13343296; doi:10.1002/bco2.70237)
Supplement: Supplementary file 1 — Table S1. Perioperative and oncological outcomes of MRI‐guided transperineal prostate biopsy between 10/2018 and 04/2024. All values are median (Interquartile range) or frequencies (%). [file BCO2-7-e70237-s002.docx]

|  |  |  |  |  |  |  |
| --- | --- | --- | --- | --- | --- | --- |
|  | **Overall,**  N = 3,336 | **caseload:**  **1-50,**  N = 1367 (41%) | **caseload: 51-100,**  N = 1005 (30%) | **caseload: 101-150,**  N = 540 (16%) | **caseload: ≥150,**  N = 424  (13%) | **p-value** |
| Procedure duration [min] | 11  (9, 16) | 13  (10, 18) | 10  (8, 15) | 10  (8, 15) | 10  (9, 15) | <0.001 |
| No. of total cores | 16.00  (15.00, 18.00) | 16.00  (15.00, 18.00) | 16.00  (15.00, 18.00) | 15.00  (15.00, 17.00) | 15.00 (14.00, 16.00) | <0.001 |
| No. of targeted cores | 4.00  (3.00, 6.00) | 4.00  (3.00, 6.00) | 4.00  (3.00, 6.00) | 3.00  (3.00, 5.00) | 3.00  (2.00, 4.00) | <0.001 |
| No. of systematic cores | 12.00 (12.00,12.00) | 12.00  (12.00, 12.00) | 12.00  (12.00, 12.00) | 12.00 (12.00,12.00) | 12.00 (12.00, 12.00) | 0.079 |
| No. of positive total cores | 1.00  (0.00, 4.00) | 1.00  (0.00, 4.00) | 1.00  (0.00, 4.00) | 1.00  (0.00, 4.00) | 1.00  (0.00, 4.00) | >0.9 |
| No. of positive targeted cores | 0.00  (0.00, 2.00) | 0.00  (0.00, 2.00) | 0.00  (0.00, 2.00) | 0.00  (0.00, 2.00) | 0.00  (0.00, 2.00) | 0.6 |
| No. of positive systematic cores | 0.00  (0.00, 2.00) | 0.00  (0.00, 2.00) | 1.00  (0.00, 2.00) | 0.00  (0.00, 2.00) | 0.00  (0.00, 3.00) | 0.6 |
| Caseload per surgeon | 64  (30, 110) | 24  (12, 37) | 73  (61, 86) | 122  (110, 135) | 185  (167, 209) | <0.001 |
| csPCa based on total cores |  |  |  |  |  | 0.8 |
| No | 1,894 (57%) | 762 (57%) | 582 (59%) | 307 (57%) | 243 (57%) |  |
| Yes | 1,410 (43%) | 585 (43%) | 412 (41%) | 233 (43%) | 180 (43%) |  |
| csPCa based on targeted cores |  |  |  |  |  | 0.6 |
| No | 2,137 (65%) | 851 (63%) | 653 (65%) | 357 (66%) | 276 (65%) |  |
| Yes | 1,170 (35%) | 495 (37%) | 344 (35%) | 183 (34%) | 148 (35%) |  |
| csPCa based on systematic cores |  |  |  |  |  | >0.9 |
| No | 2,150 (65%) | 879 (65%) | 649 (65%) | 353 (65%) | 269 (63%) |  |
| Yes | 1,157 (35%) | 469 (35%) | 347 (35%) | 186 (35%) | 155 (37%) |  |

**Abbreviations:** No.=Number; csPCa=Clinically significant prostate cancer; MRI=Magnetic-resonance imaging
