# Supplementary material for: Procedure efficiency and diagnostic outcomes during the learning curve of transperineal MRI‐guided prostate biopsy
Source: BJUI Compass. 2026 Jul 8;7(7):e70237. doi: 10.1002/bco2.70237 (PMC13343296; doi:10.1002/bco2.70237)
Supplement: Supplementary file 2 — Table S2. Separate univariable and multivariable logistic regression models investigating the predictor status for detection of clinically significant prostate cancer according to caseload among 3336 cases treated with MRI‐guided transperineal prostate biopsy stratified according to index PI‐RADS lesion (PI‐RADS 3, PI‐RADS 4, PI‐RADS 5). [file BCO2-7-e70237-s001.docx]

|  |  |  |  |  |  |  |  |  |  |
| --- | --- | --- | --- | --- | --- | --- | --- | --- | --- |
| PI-RADS 3 | **Univariable** | | | |  | **Multivariable*** | | | |
|  | **Odds Ratio** | **2.5 %** | **97.5 %** | **p-value** |  | **Odds Ratio** | **2.5 %** | **97.5 %** | **p-value** |
| Caseload per surgeon  1-50 | Ref. |  |  |  |  | Ref. |  |  |  |
| 51-100 | 0.9838 | 0.6867 | 1.4055 | 0.9287 |  | 0.9834 | 0.6664 | 1.4478 | 0.9326 |
| 101-150 | 1.1715 | 0.7642 | 1.7754 | 0.4607 |  | 1.3832 | 0.8685 | 2.1840 | 0.1671 |
| ≥151 | 1.0208 | 0.6253 | 1.6282 | 0.9327 |  | 1.0356 | 0.6003 | 1.7519 | 0.8979 |
|  | | | | | | | | | |
| PI-RADS 4 | **Univariable** | | | |  | **Multivariable*** | | | |
|  | **Odds Ratio** | **2.5 %** | **97.5 %** | **p-value** |  | **Odds Ratio** | **2.5 %** | **97.5 %** | **p-value** |
| Caseload per surgeon  1-50 | Ref. |  |  |  |  | Ref. |  |  |  |
| 51-100 | 1.0272 | 0.7942 | 1.3282 | 0.8380 |  | 1.1327 | 0.8539 | 1.5032 | 0.3876 |
| 101-150 | 1.1422 | 0.8347 | 1.5625 | 0.4054 |  | 1.2067 | 0.8549 | 1.7040 | 0.2853 |
| ≥151 | 1.0694 | 0.7667 | 1.4899 | 0.6917 |  | 1.0112 | 0.6951 | 1.4697 | 0.9534 |
|  | | | | | | | | | |
| PI-RADS 5 | **Univariable** | | | |  | **Multivariable*** | | | |
|  | **Odds Ratio** | **2.5 %** | **97.5 %** | **p-value** |  | **Odds Ratio** | **2.5 %** | **97.5 %** | **p-value** |
| Caseload per surgeon  1-50 | Ref. |  |  |  |  | Ref. |  |  |  |
| 51-100 | 0.9237 | 0.6391 | 1.3392 | 0.6739 |  | 0.9194 | 0.6068 | 1.3974 | 0.6926 |
| 101-150 | 0.8408 | 0.5395 | 1.3239 | 0.4479 |  | 0.7754 | 0.4734 | 1.2798 | 0.3152 |
| ≥151 | 1.0535 | 0.6297 | 1.8075 | 0.8458 |  | 0.7083 | 0.3811 | 1.3351 | 0.2793 |

*Adjusted for: age, digital rectal examination, number of PI-RADS lesion, PSA at biopsy, prostate volume, history of prostate cancer, experience in transrectal prostate biopsy
